# Explicating heterogeneity of complex traits has strong potential for improving GWAS efficiency

**DOI:** 10.1038/srep35390

**Published:** 2016-10-14

**Authors:** Alexander M. Kulminski, Yury Loika, Irina Culminskaya, Konstantin G. Arbeev, Svetlana V. Ukraintseva, Eric Stallard, Anatoliy I. Yashin

**Affiliations:** 1 Biodemography of Aging Research Unit, Social Science Research Institute, Duke University, Durham, NC 27708-0408, USA

## Abstract

Common strategy of genome-wide association studies (GWAS) relying on large samples faces difficulties, which raise concerns that GWAS have exhausted their potential, particularly for complex traits. Here, we examine the efficiency of the traditional sample-size-centered strategy in GWAS of these traits, and its potential for improvement. The paper focuses on the results of the four largest GWAS meta-analyses of body mass index (BMI) and lipids. We show that just increasing sample size may not make p-values of genetic effects in large (N > 100,000) samples smaller but can make them larger. The efficiency of these GWAS, defined as ratio of the log-transformed p-value to the sample size, in larger samples was larger than in smaller samples for a small fraction of loci. These results emphasize the important role of heterogeneity in genetic associations with complex traits such as BMI and lipids. They highlight the substantial potential for improving GWAS by explicating this role (affecting 11–79% of loci in the selected GWAS), especially the effects of biodemographic processes, which are heavily underexplored in current GWAS and which are important sources of heterogeneity in the various study populations. Further progress in this direction is crucial for efficient use of genetic discoveries in health care.

New insights into genetic predisposition to diseases, related traits, and survival could substantially contribute to improvement of healthspan, particularly in aging populations in developed countries^1^,^2^,^3^. Genome-wide association studies (GWAS) have been thought to accelerate the progress in this endeavor.

Despite the apparent GWAS successes^4^, it is recognized that the common GWAS strategy faces serious difficulties, especially in the case of complex traits^5^,^6^,^7^. For example, one difficulty is the problem of the effect sizes: GWAS typically report associations with modest effects and even smaller effects are expected to be detected according to the infinitesimal hypothesis^5^. This difficulty is related to the problem of missing heritability^8^. Another difficulty is the problem of non-replication of genetic associations with complex traits^9^,^10^,^11^. These difficulties raise concerns that GWAS have exhausted their potential for complex traits and new strategies are needed^6^.

Various strategies for overcoming GWAS difficulties have been discussed. One broad category of strategies emphasizes the role of genetic factors such as rare variants, epigenetic modifications, miRNA, etc.^6^. Another category highlights the critical role of inherent heterogeneity of complex traits, heterogeneity which encompasses the complexity of endogenous and exogenous mechanisms predisposing to these traits^5^,^7^,^12^,^13^,^14^. The stochastic component in genetic susceptibility to complex traits may be another important factor^15^.

These strategies may or may not fit those which are widespread in current GWAS. For example, GWAS rely heavily on “the benefits of the large sample sizes achievable through collaboration”^16^ for detecting risk alleles of complex traits. This strategy makes perfect sense if one assumes that genetic susceptibility to a trait of interest is homogeneous, i.e., the same genetic variant, or, more broadly, biological process, predispose to this trait in different people in a given population. Evolutionary biology provides, however, little support to this hypothesis; rather, it argues that genetic predisposition to complex traits is inherently heterogeneous^17^,^18^ (see more details in the Discussion section “**Why and how to improve the traditional GWAS strategy in case of complex traits?**”). Then, the analyses of genetic predisposition to heterogeneous traits (as opposite to homogeneous traits defined above) relying on increasing sample size become problematic because “increasing the size of human disease cohorts is likely only to scale the heterogeneity in parallel”^7^.

In this paper, we examine the potential for improving the traditional GWAS strategy, which relies on increasing the sample size, in the case of inherently heterogeneous traits. The paper focuses on the results of the largest GWAS meta-analyses, so far. They include studies of lipids comprising nearly 100,000 individuals^19^ and nearly 188,000 individuals^16^, and studies of body mass index (BMI) comprising nearly 250,000 individuals^20^ and nearly 320,000 individuals^21^ of European descent.

## Results

### Estimates in GWAS with larger and smaller samples

**Figure 1:**
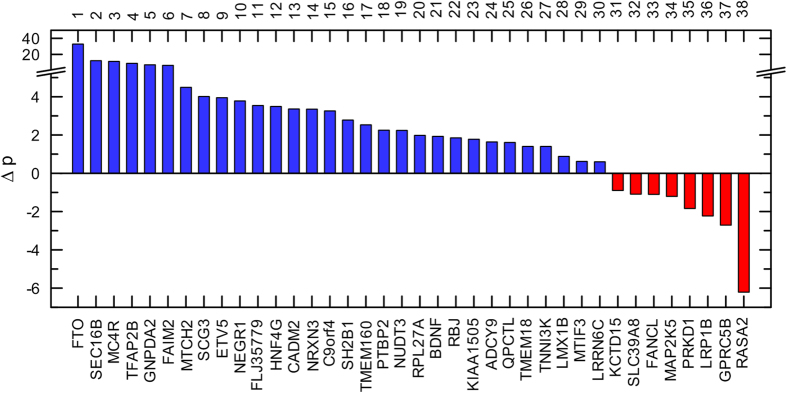
P-value gain in the BMI GWAS. The y-axis shows the difference in log-transformed p-values reported in larger (p_2015_)^21^ and smaller (p_2010_)^20^ GWAS, i.e., ∆*p* = −(log_10_(*p*_2015_) − log_10_(*p*_2010_)). The lower x-axis denotes 38 overlapping loci reported in these studies. Red color shows loci for which p-values were larger in the larger sample compared to the smaller one. The upper x-axis shows the order of loci. Other details and numerical estimates are given in Supplementary Table 1.

To examine the efficiency of the traditional GWAS strategy, we first compared p-values between larger and smaller samples (see “**Methods**”). Figure 1 shows that BMI p-values in the larger sample^21^ (N~320 K) were larger than in the smaller sample^20^ (N~240 K) for 21.1%, i.e., 8 of 38 loci. For 11 additional loci, we observed minor change in p-values with their decrease by just two orders of magnitude despite increasing the sample size by about 33%. Therefore, the BMI p-values were either larger or had minor decrease (within two orders of magnitude) for 50% of loci.

For 13 of 38 SNPs (34.2%) the BMI p-values were either larger (5 loci) or had minor decrease (8 loci) in the larger sample compared to the smaller sample in the same study^20^ despite nearly two-fold difference in the sample sizes (Supplementary Figure 1). In the other study^21^, the BMI p-values in the larger sample were smaller than in the smaller sample by at least four orders of magnitude (Supplementary Figure 2) due to a nearly four-fold sample size difference between them (Supplementary Table 3).

For the lipid meta-analyses, the p-values were either larger (5 loci) or had minor decrease (4 loci within two orders of magnitude) for nine of 76 loci (11.8%) in the larger study^16^ (N~188 K) than in the smaller study^19^ (N~100 K) (Supplementary Figure 3).

### Relative efficiency of GWAS

At first glance, the results in Supplementary Figure 2 appear to support the benefits of large samples in GWAS. The analysis presented in Fig. 1 and Supplementary Figures 1 and 3 provide less support, however. To gain further insights on the efficiency of GWAS, we evaluated the relative efficiency *ρ* (see Methods, “**Efficiency measure**”) of GWAS in the same pairs of larger and smaller samples as above.

**Figure 2:**
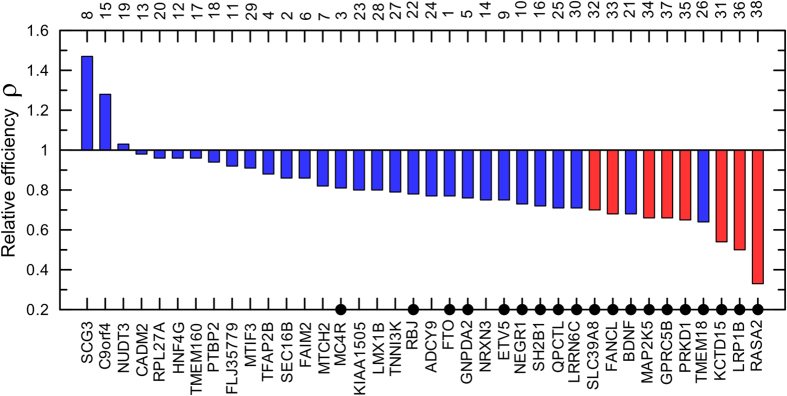
Relative efficiency of the BMI GWAS. The relative efficiency indicates the gain in log-transformed p-value per person in the larger GWAS^21^ compared to the smaller GWAS^20^. The lower x-axis denotes 38 overlapping loci reported in these studies. Red color shows loci for which p-values were larger in the larger sample compared to the smaller sample in Fig. 1. Numbers on the upper x-axis show the order of loci in Fig. 1. Filled dots denote loci for which deviation from *ρ* = 1 attained statistical significance. Other details and numerical estimates are given in Supplementary Table 1.

Figure 2 shows that the relative efficiency of the BMI GWAS in refs 21,20 varied between 0.3 and 1.5. Higher efficiency in the larger sample than in the smaller sample (i.e., *ρ* > 1) was found for 3 of 38 loci (7.9%). However, the estimates of *ρ* for these three loci did not attain statistical significance (Fig. 2 and Supplementary Note 1). The relative efficiency *ρ* was significantly smaller than 1 for 19 of 38 loci (50%). Figure 2 shows lower efficiency not only for loci with larger p-values in the larger sample (Fig. 1) but also for those with modestly smaller p-values.

**Figure 3:**
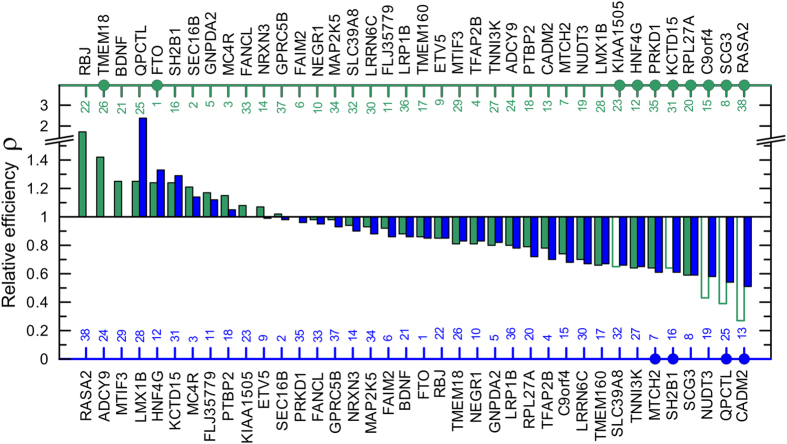
Relative efficiency in two sets of GWAS of BMI shown by different color. Blue color shows the relative efficiency ρ for the entire sample vs. Metabochip in ref. 21 for loci shown on the lower x-axis. Green color shows the relative efficiency ρ for the entire sample vs. Stage 1 in ref. 20 for loci shown on the upper x-axis. Unfilled green bars show loci for which p-values were larger in the larger sample compared to the smaller sample in Fig. 1. Numbers on the upper and lower x-axes show the order of loci in Fig. 1. Filled dots denote loci for which deviation from *ρ* = 1 attained statistical significance. Other details and numerical estimates are given in Supplementary Tables 2 and 3.

Figure 3 shows the relative efficiency of the BMI GWAS in the selected samples in each study. For GWAS from ref. 21, the efficiency in the larger sample (the entire sample, N~320 K) was higher than that in the smaller sample (Metabochip, N~88 K) for 7 of 35 loci (20.0%). For all 7 loci the estimates of *ρ* did not attain significance (Fig. 3 and Supplementary Note 1). The relative efficiency *ρ* was significantly smaller than 1 for 4 of 35 loci (11.4%). For GWAS in ref. 20, the efficiency in the larger sample (N~250 K) was higher than the efficiency in the smaller sample (Stage 1, N~123 K) for 12 of 38 loci (31.6%). For two of them (TMEM18 and FTO) *ρ* was significantly larger than 1. For 8 of 38 loci (21.1%) *ρ* was significantly smaller than 1 (Fig. 3 and Supplementary Note 1).

Figure 3 shows that the relative efficiency of the analyses became consistently higher with increasing the sample size in refs 20,21 only for one BMI locus (MC4R).

**Figure 4:**
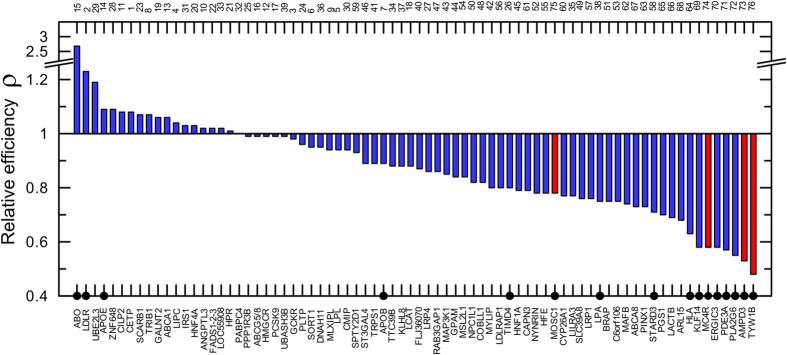
Relative efficiency of the lipid GWAS. The relative efficiency indicates the gain in log-transformed p-value per person in the larger GWAS^16^ compared the smaller GWAS^19^. The lower x-axis denotes 76 overlapping loci reported in these studies. Red color shows loci for which p-values were larger in the larger sample compared to the smaller sample in Supplementary Figure 3. Numbers on the upper x-axis show the order of loci in Supplementary Figure 3. Filled dots denote loci for which deviation from *ρ* = 1 attained statistical significance. Other details and numerical estimates are given in Supplementary Table 4.

Figure 4 shows that the relative efficiency of GWAS of lipids in the larger sample^16^ compared to the smaller sample^19^ was higher for 18 of 76 loci (23.7%). For three of them (ABO, LDLR, and APOE) *ρ* was significantly larger than 1. For 13 of 76 loci (17.1%) *ρ* was significantly smaller than 1.

### Potential for improving the efficiency of GWAS

Improving the efficiency of the analyses implies that fewer people are needed to achieve the same result as in the case of conventional (unimproved) efficiency. One potential source is improving the efficiency of the analyses in larger samples (characterized by *ξ*_1_) for loci with *ρ* = *ξ*_1_/*ξ*_2_ < 1 because *ξ*_1_ < *ξ*_2_ in this case.

It can be argued, however, that the situation with *ρ* < 1 can be common in follow up studies with larger samples because of the winner’s curse effect^22^. The winner’s curse hypothesis was adapted from the auction theory implying that in an auction the winner tends to overpay. In genetic association studies, it may characterize an ascertainment bias due to focusing on upwardly biased effect sizes capable to yield significant associations in the discovery studies. This hypothesis was introduced in pre-GWAS era to explain the lack of replication of genetic effects in the follow up studies which were of substantially smaller sample sizes compared to GWAS with N > 100 K individuals considered in the current paper. These large samples substantially weaken arguments on a pivotal role of the winner’s course effect in the situation with *ρ* < 1.

More importantly, however, is that in biology any inferences should be considered from the viewpoint of evolution (see the Discussion section “**Why and how to improve the traditional GWAS strategy in case of complex traits?**”). The role of evolution in the winner’s course effect is, however, unclear, particularly in the selected BMI and lipid GWAS.

The evolutionary theory suggests that genetic predisposition to complex traits should be inherently heterogeneous. Deviation from *ρ* = 1 is consistent with heterogeneity in genetic effects between two samples (see the Analysis section “**Efficiency measure**”). Accordingly, the situation with *ρ* < 1 can be not only due to the winner’s course effect but it can naturally be due to heterogeneity.

To quantify the potential for improving the efficiency in this case one needs to determine cut off for *ρ*. It is not entirely clear, however, how to do that. One approach is to select the cut off based on significance of *ρ* < 1 for specific loci (Figs 2). This approach implicitly assumes that the non-significant estimates for loci with *ρ* < 1 are likely the result of stochastic realization. Given insights from the evolutionary theory, non-significant estimates for loci with *ρ* < 1 may not necessarily be due to stochasticity but they can also be due to heterogeneity. Then, the less conservative approach would be to select the cut off based on cost/benefit reasoning.

For example, assuming a 90% relative efficiency of GWAS (i.e., *ρ* = 0.9) implies that the efficiency *ξ*_1_ (i.e., the ratio of the log-transformed p-value to the sample size) was larger by 10% in a larger sample than in a smaller one. This means that the efficiency of the analysis in the larger sample is 10% smaller than in the smaller sample. This is equivalent to underusing 10% of the available sample that in large samples with N > 100 K leads to underusing information on more than 10 K people. Therefore, by improving the efficiency *ξ*_1_, one can use 10% smaller sample in this case to achieve the same result as in the case of conventional (unimproved) efficiency. Table 1 shows that this improvement would also affect a large proportion of loci which, for 90% efficiency, ranges from 52.6% to 73.7%. Thus, given cost (in terms of investment in 10% increase of the sample size in this case) and benefits (in terms of using non-increased sample but conducting more rigorous analyses) one could decide which strategy would be beneficial in a specific situation.

The situation with *ρ* = *ξ*_1_/*ξ*_2_ > 1 indicates that there is also potential for improving the efficiency but in smaller samples characterized by *ξ*_2_. The same approaches to determine cut off for *ρ* as in the case of *ρ* < 1 (see above) hold here.

Because deviation from *ρ* = 1 (i.e., either *ρ* < 1 or *ρ* > 1) is consistent with heterogeneity, the potential for improvement can likely be in exploring more rigorous approaches to explicate an inherent heterogeneity in genetic associations with complex traits which remains after handling cross-sample heterogeneity using genomic methods used in the referenced GWAS meta-analyses (see the Discussion section “**Why and how to improve the traditional GWAS strategy in case of complex traits?**”).

Based on statistical tests, deviation from *ρ* = 1 was significant for: (i) 19 of 38 loci (50%) for the BMI GWAS in refs 21,20 (Fig. 2), (ii) 4 of 35 loci (11.4%) for the BMI GWAS in ref. 21 (Fig. 3, blue color), (iii) 10 of 38 loci (26.3%) for the BMI GWAS in ref. 20 (Fig. 3, green color), and (iv) 16 of 76 loci (21.1%) for GWAS of lipids in refs 16,19 (Fig. 4). According to this approach, significant deviation is observed for 11% to 50% of loci. Based on the cost/benefit approach, deviation from *ρ* = 1 by 10% (i.e., 1.1 < *ρ* < 0.9) is observed for 62% to 79% of loci (Table 1). Thus, these results indicate a substantial potential for improvement which may affect 11% (the most conservative estimate) to 79% (the less conservative estimate) of loci in the selected four GWAS.

## Discussion

GWAS often relies on “the benefits of the large sample sizes”^16^ for detecting risk alleles of complex traits. Conversely, it is also argued that the traditional GWAS strategy merely relying on increasing the sample size is problematic because of the inherent heterogeneity of complex traits^7^. As a result, common GWAS may substantially underuse the available resources. In this paper, we examined the efficiency of GWAS of lipids^16^,^19^ and BMI^20^,^21^, which are the largest GWAS meta-analyses so far, and highlighted the potential for improving GWAS efficiency.

Next, we emphasize three important results, which support the strong potential for improving the efficiency of GWAS of complex, inherently heterogeneous traits.

First, our analyses show that the estimates of the significance of genetic effects may decrease with increasing sample size, i.e., p-values become larger in larger samples compared to smaller samples. For example, the significance of the BMI estimates was larger for 21% of loci in the larger sample (N~320 K) than in the smaller sample (N~240 K) (Fig. 1). Importantly, the large sample sizes of these “larger” and “smaller” samples offset the problem of stochastic variation in p-values, which is more likely in small samples.

Second, the analyses of samples of larger and smaller sizes showed that the efficiency of GWAS was larger in the larger samples for a small fraction of loci ranging from 7.9% (3 of 38 loci) for the BMI GWAS in refs 21,20 (Fig. 2) to 31.6% of loci (12 of 38) for the BMI GWAS in two samples in ref. 20 (Fig. 3, green color). The benefit of larger samples was supported by statistical significance for 2 loci for the BMI GWAS (Fig. 3, green color) and for 3 loci in GWAS of lipids (Fig. 4).

Third, consistent increase of the relative efficiency was found for only one BMI locus.

These results lead to three important conclusions. First, increasing the sample size of the study population in genetic analyses of complex traits does not necessarily decrease the estimates of the significance of genetic effects (p-values) but can actually increase p-values. Second, our results support the substantial role of heterogeneity in genetic predisposition to complex traits such as BMI and lipids. Importantly, this is an inherent (trait-specific) heterogeneity due to the elusive role of evolution in these traits (see below), which remains after handling cross-study heterogeneity using genomics methods in the referenced GWAS. Third, the results highlight the substantial potential for improving GWAS by explicating this inherent heterogeneity that may affect 11% (the most conservative estimate) to 79% (the less conservative estimate) of loci in the selected four GWAS.

Why and how to improve the traditional GWAS strategy in case of complex traits ? A key argument for increasing sample size in GWAS of traits with moderate and small effect sizes is to have sufficient statistical power to detect genetic effects. This is because low power decreases the likelihood that a significant association actually reflects “a true effect”^23^. Accordingly, a key hypothesis behind this argument is that “a true” genetic effect on a trait exists. The discipline of biology argues that “nothing in biology makes sense except in the light of evolution”^24^. Therefore, understanding the role of evolution in complex traits is critical in studies of genetic (i.e., biological) predisposition to these traits.

Evolutionary biology, epidemiology, and aging research argue that: (i) environmental exposures in modern societies are dramatically different than those in the past and (ii) complex traits may not be subject to direct evolutionary selection^17^,^18^,^25^. These factors imply that genetic variants may not have a wide norm of reaction for complex traits^24^. Furthermore, if the strategic goal is to improve human well-being, healthspan, and lifespan^3^, GWAS necessarily face a need to deal with a special class of traits, called age-related disease traits, i.e., traits, which are characteristic of the elderly people in modern societies. These are complex polygenic traits. Unlike the other complex traits, age-related disease traits have three important aspects. First, from the evolutionary point of view they are a relatively new massive phenomenon. This is because, for example, in 1840 the world record of mean lifespan for women was about 45 years^26^ implying that about half of the population did not survive to older ages where incidence of the age-related disease traits sharply increases. Second, they are characteristic of the post-reproductive period where selection pressure is not as strong as at the reproductive period. Third, these traits appear in late life whereas genes are transmitted from parents at conception, i.e., these events are separated by a large portion of the individuals’ life. It can be argued that refocusing from the genetics of age-related diseases to their precursors (often called endophenotypes), which are characteristic for reproductive age, could benefit the analyses. However, the role of evolution in endophenotypes is also elusive because genes regulating endophenotypes have not been directly selected against or in favor of their pathological dysregulation causing age-related diseases.

Sensitivity of genetic effects to the environment due to narrow norm of reaction to complex traits and specific properties of age-related traits weaken the conceptual basis of the hypothesis of “true” genetic effects on complex traits^5^,^17^ and strengthen the hypothesis of complex roles of the same genetic variants in the same trait^27^. The latter implies genetic heterogeneity. As a result, the common GWAS strategy relying mostly on increasing the sample size becomes problematic in case of complex traits.

A key to improve the efficiency of the analyses of genetic predisposition to complex traits without increasing sample size is to better handle heterogeneity. However, heterogeneity is the result of various processes that requires better understanding diversity of its sources. Common sources include: (i) processes associated with evolutionarily selected genetic patterns in populations, (ii) complex etiologies of human phenotypes, (iii) environmental influences, and (iv) age-related heterogeneity attributed to the elusive role of evolution in the development of age-related phenotypes. Clearly, different sources of heterogeneity require different strategies to work with. Common practice in GWAS is to handle heterogeneity attributed to population structures, using, for example, methods of principal component analysis^28^. These methods may efficiently address the first source of heterogeneity especially in young populations with no substantial survival selection. The second source can be addressed by refining the architecture of complex traits and by examining more homogeneous sub-phenotypes^6^,^7^. The third source requires addressing gene by environment interactions. The fourth source requires analyses of age-related changes in an organism over its life course and in populations (which takes into account age, cohort, and survival effects), as well as the analyses of mechanistic pathways from genes to downstream phenotypes through endophenotypes^17^,^29^,^30^.

Evidences of the importance of the role of age-related heterogeneity in genetic associations are accumulating in the field. For example, the analyses highlighted the role of age in genetic regulation of BMI^31^, sensitivity of the effects of longevity alleles to birth cohorts^12^,^32^, sensitivity of genetic associations with lipids to chronological age^33^,^34^, and changes in the allele frequencies with age^35^,^36^.

Thus, refocusing the analyses to a realistic concept of complex, inherently heterogeneous traits reflecting elusive role of evolution in these traits, has substantial potential for improving the efficiency of GWAS. Parameter of the relative efficiency ρ could help in prioritizing SNPs for more comprehensive analyses. This is crucial not only for a better understanding of the genetic influences on these traits, but also for efficient use of genetic discoveries in health care.

## Methods

### Selection of SNPs representing loci associated with BMI and lipids

We identified SNPs for 38 loci associated with BMI which were reported in refs 20,21 (Supplementary Table 1). These loci were selected for the analyses.

We also selected SNPs for 76 loci associated with lipids which were reported in refs 19,16 (Supplementary Table 4), as described below. We considered SNPs with the best associations reported in ref. 16 with one of the lipid traits, which include total cholesterol, low-density lipoprotein cholesterol, high-density lipoprotein cholesterol, and triglycerides.

### Analysis

To examine the efficiency of GWAS and its potential for improvement, we used two strategies. First, we compared the significance of the estimates (p-values) in larger and smaller samples. Second, we used a measure of the efficiency as detailed in the subsection below.

We compared the results from the BMI meta-analyses^20^,^21^ and the lipid meta-analyses^16^,^19^ separately. The focus was on comparative analyses of the results in larger and smaller samples of individuals of European descent.

The results for the selected 38 BMI loci were presented in ref. 20 for the entire sample (N~250 K individuals) and for two subsamples, Stage 1 (N~123 K) and Stage 2 (N~125 K). The results for these loci were presented in ref. 21 for the entire sample (N~320 K) as well as for two subsamples, Stages 1+2 (N~233 K) and Metabochip (N~88 K). We compared the results between larger and smaller samples. For all loci except one (RASA2) the sample size was larger in ref. 21 than in ref. 20. For RASA2, we used the results from ref. 20 to represent the larger sample. Comparative analyses within ref. 20 were focused on the entire sample and Stage 1. All 38 selected loci were used in these analyses (Supplementary Table 2). Comparative analyses within ref. 21 were focused on the entire sample and Metabochip. We used 35 of 38 loci for these analyses because of the lack of estimates for three of them on Metabochip (RASA2, ADCY9, and MTIF3, see Supplementary Table 3).

The results for the lipid loci were presented for the entire samples in each study, i.e., ref. 16 (N~188 K) and ref. 19 (N~100 K). Because for some SNPs the sample size in the analyses in ref. 16 was actually about the same as in ref. 19, we selected only those 76 loci for which sample size in ref. 19 was larger by at least 10,000 individuals than in ref. 16.

### Efficiency measure

To better characterize the potential for improvement of GWAS, we derived a measure of the efficiency of GWAS meta-analyses (Supplementary Note 1 and Refs 37, 38, 39). This measure represents the ratio of the log-transformed probability of the effect *b* to the sample of size *N*, i.e., *ξ* = −log_10_(*p*)/N. The efficiency measure can be interpreted as the log-transformed *p*-value per unit observation, i.e., per person in this case. The estimate of the relative efficiency in two samples was defined as *ρ* = *ξ*_1_/*ξ*_2_. We used *ξ*_1_ for the larger sample and *ξ*_2_ for the smaller one. For a given trait-loci association, the relative efficiency *ρ* shows which sample had larger log-transformed p-value per unit (person). Then, if the efficiency *ξ* is larger in smaller sample than in larger sample then the analyses in larger sample are less efficient than in smaller sample.

The relative efficiency *ρ* is also a convenient characteristic of homogeneity/heterogeneity in genetic susceptibility to a given trait (see Supplementary Note 1). The assumption of a homogeneous genetic effect for a given trait implies that *ρ* = *ξ*_1_/*ξ*_2_ = 1. Deviation from *ρ* = 1 is consistent with heterogeneity in genetic effects between two samples.

## Additional Information

**How to cite this article**: Kulminski, A. M. *et al.* Explicating heterogeneity of complex traits has strong potential for improving GWAS efficiency. *Sci. Rep.* **6**, 35390; doi: 10.1038/srep35390 (2016).

## Supplementary Material

Supplementary Information

## Figures and Tables

**Table 1:** Proportions of loci for selected levels of the relative efficiency of GWAS of BMI and lipids.

Base	*ρ* < 0.9		*ρ* < 0.8		1.1 < *ρ* < 0.9		1.2 < *ρ* < 0.8		Compared samples	Figure
	N	%	N	%	N	%	N	%		
38	28	73.7	21	55.3	30	78.9	23	60.5	ref. 21 all vs. ref. 20 all	Fig. 2
35	22	62.9	14	40.0	27	77.1	17	48.6	ref. 21 all vs. ref. 21 Metabochip	Fig. 3
38	20	52.6	13	34.2	29	76.3	20	52.6	ref. 20 all vs. ref. 20 Stage 1	Fig. 3
76	44	57.9	27	35.5	47	61.8	29	38.2	ref. 16 all vs. ref. 19 all	Fig. 4

N denotes the number of loci with the specified relative efficiency *ρ*.

Column “Base” shows the number of loci selected for the analysis.

Parameter *ρ* = *ξ*_*1*_/*ξ*_*2*_ is the relative efficiency indicating as the gain in the log-transformed p-value per person in a larger sample compared to the smaller sample (given in column “Compared samples”).
