# Supplementary material for: Explicating heterogeneity of complex traits has strong potential for improving GWAS efficiency
Source: Sci Rep. 2016 Oct 14;6:35390. doi: 10.1038/srep35390 (PMC5064392; doi:10.1038/srep35390)
Supplement: Supplementary Information [file srep35390-s1.pdf]

Explicating heterogeneity of complex traits has strong potential for improving GWAS efficiency

Alexander M. Kulminski*, Yury Loika, Irina Culminskaya, Konstantin G. Arbeev, Svetlana V. Ukraintseva, Eric Stallard, and Anatoliy I. Yashin

Biodemography of Aging Research Unit, Social Science Research Institute, Duke University, Durham, NC 27708-0408, USA.

* Alexander.Kulminski@duke.edu

Running title: **GWAS and heterogeneity**

1. Supplementary Notes

Supplementary Note 1

Asymptotic expression for the efficiency ξ for a normally distributed random variable and homogeneity/heterogeneity of targeted effects

Consider two estimates of the same regression equation from two separate sample populations, P_1 and P_2, of size N_1 and N_2, where $N_1 > N_2$; denote the respective estimates of two regression effects as $\hat{b}_1$ and $\hat{b}_2$. Consider the null hypothesis: (H0) that $b_1 = b_2 = b_0$, where b_0 is some prespecified value which, for simplicity, we assume to be $b_0 = 0$. Because $\hat{b}_1$ and $\hat{b}_2$ are sample estimates, we extend the null hypothesis by assuming that they are drawn from closely related normal populations having variance σ_0^2/N_i , $i = 1,2$, where σ_0^2 is a common parameter termed the "unit variance" and where the division by N_i follows from well-known properties of regression estimators, see p.226 in Ref. [37]. The assumption that σ_0^2 is a common parameter implies that the variances of the predictors in the regression equations are equal in the two samples, which will be true if the samples are drawn from a common parent population using the same sampling methods.

Define the t-transformation as $t_i = b_i\sqrt{N_i}/\sigma_0\,, i = 1,2$. It follows from the above assumptions that the estimator $\hat{t}_i$ is distributed as a standard normal variate with variance 1. The probability that a hypothetical estimate, say $\tilde{t}_i$, is larger than $\hat{t}_i$ is then given by:

$$p(\hat{t}_i) \equiv Pr[\tilde{t}_i > \hat{t}_i] = \int_{\hat{t}_i}^{\infty} f(\tilde{t})d\tilde{t}\ , \qquad (1)$$

where $f(\tilde{t}) = \frac{1}{\sqrt{2\pi}} exp(\tilde{t}^2/2)$ is the standard normal probability density function. Equation (1) is the probability of an observed effect of size $\hat{t}_i$ or larger in standardized units in a sample of size N_i, assuming that the null hypothesis is true. This is the expression for the quantity typically reported as the p-value for a given effect in GWAS.

For simplicity, we consider only the typical GWAS case where $\hat{t}_i \gg 0$. Hence, we focus on the upper tail probability of the standard normal distribution, i.e., the Q-function:

$$p(\hat{t}_i) \equiv Q(\hat{t}_i) = \frac{1}{\sqrt{2\pi}} \int_{\hat{t}_i}^{\infty} exp(-\tilde{t}^2/2)d\tilde{t}\ , \qquad (2)$$

which can be approximated as: [38]

$$p(\hat{t}_i) \approx \frac{1}{12} exp\left(-\frac{1}{2}\hat{t}_i^2\right) + \frac{1}{4} exp\left(-\frac{2}{3}\hat{t}_i^2\right) \approx \alpha \cdot exp(-\beta \cdot \hat{t}_i^2), \qquad (3)$$

with pseudo-parameters $\alpha = 0.0559$ and $\beta = 0.5030$ estimated numerically by minimizing the squared relative differences between the probability density function for the standard normal distribution and the approximating solution for $Q(\hat{t}_i)$ within the typical for GWAS range $4 \le \hat{t}_i \le 20$.

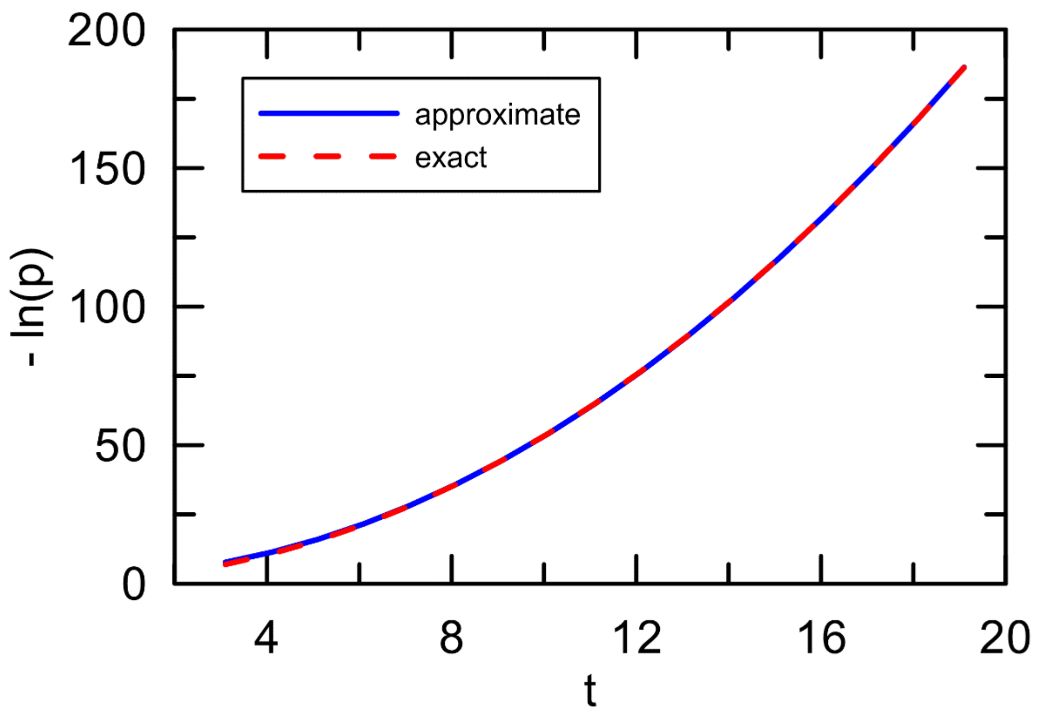

The above figure shows that the second approximation to the *Q*-function is virtually indistinguishable from the original *Q*-function on a logarithmic scale over the indicated range [4, 20].

Hence, taking the natural logarithm of (3), changing sign, replacing the parameters by their estimators, substituting for $\hat{t}_i^2$, and dividing by N_i, we obtain for population P_i:

$$\hat{\xi}_i \equiv -\frac{\ln p(\hat{b}_i)}{N_i} \approx -\frac{\ln \alpha}{N_i} + \frac{\beta\, \hat{t}_i^2}{N_i} \approx \frac{\beta\, \hat{b}_i^2}{\sigma_0^2}, \tag{4}$$

where, except for the scaling parameter ln(10) in the denominator for the change from natural to base 10 logarithms, ξ_i is the efficiency parameter introduced in the "**Efficiency measure**" section in the main text and $\hat{\xi}_i$ is its estimator. Thus, the efficiency ξ_i represents the ratio of the log-transformed probability of the effect b_i to the sample of size N_i. Equivalently, the efficiency can be interpreted as the log-transformed *p*-value per unit observation, i.e., per person in this case.

If the null hypothesis (H0) is true, then $p(\hat{b}_i)$ follows the uniform distribution over the unit interval [0, 1], independent of sample size N_i, and $\hat{\xi}_i$ follows the exponential distribution over [0, ∞) with mean $1/N_i$.

Small *p*-values are generally interpreted as providing evidence against the null hypothesis, leading to consideration of two alternative hypotheses: (H1) $b_1 = b_2 = b_{12} > b_0$; and (H2) $b_1 > b_2 > b_0$ or $b_2 > b_1 > b_0$; where H1 implies homogeneity and H2 implies heterogeneity of the targeted effect. Under H1, $\xi_1 = \xi_2$ and the estimated relative efficiency $\hat{\rho} = \hat{\xi}_1/\hat{\xi}_2$ is approximately 1. Under H2, $\xi_1 \neq \xi_2$ and the estimated relative efficiency almost always differs from 1.

The variance of $\hat{\rho}$, under an extension of H1 or H2 for which each $\hat{b}_i$, $i = 1,2$, is assumed to be drawn from a normal distribution having mean b_i and variance σ_0^2/N_i, $i = 1,2$, can be approximated using the delta method (e.g., Volume 1, p.232 in Ref. [39]).

$$var(\hat{\rho}) = \left\{\frac{E(\hat{\xi}_1)}{E(\hat{\xi}_2)}\right\}^2 \left\{\frac{var(\hat{\xi}_1)}{E^2(\hat{\xi}_1)} + \frac{var(\hat{\xi}_2)}{E^2(\hat{\xi}_2)}\right\}, \quad (5)$$

where $E(\cdot)$ denotes expectation.

Using the first approximation in eqn. (4) to introduce $\hat{t}_i^2$, which follows the 1 d.f. noncentral chi-squared distribution with $E(\hat{t}_i^2) = t_i^2 + 1$ and $var(\hat{t}_i^2) = 4(t_i^2 + 0.5)$, we obtain for large $\hat{t}_i$, i.e., $\hat{t}_i^2 \gg 1$, the following asymptotic result

$$var(\hat{\rho}) \to 4\rho^2 \left\{\frac{1}{t_1^2} + \frac{1}{t_2^2}\right\}. \quad (6)$$

Eqn. (6) can be approximated by replacing the parameters ρ and t_i with their estimators $\hat{\rho}$ and $\hat{t}_i$, both of which can be computed from $p(\hat{b}_i)$ using eqns. (3) and (4). Also since H1 implies that $t_i^2 = b_{12}^2 N_i/\sigma_0^2$, $i = 1,2$, eqn (6) can be rewritten as:

$$var(\hat{\rho}) \to \frac{4\rho^2\sigma_0^2}{b_{12}^2} \left\{\frac{1}{N_1} + \frac{1}{N_2}\right\}, \quad (7)$$

an expression which goes to zero as N_1 and N_2 both go to infinity.

Expressions (5-7) were derived based on the assumption of independence of variables $\hat{\xi}_1$ and $\hat{\xi}_2$, whereas all samples used in our analyses were not independent. To generalize these expressions to this case, we included correlation term (e.g., Volume 1, p.232 in Ref. [39])

$$var(\hat{\rho}) = \left\{\frac{E(\hat{\xi}_1)}{E(\hat{\xi}_2)}\right\}^2 \left\{\frac{var(\hat{\xi}_1)}{E^2(\hat{\xi}_1)} + \frac{var(\hat{\xi}_2)}{E^2(\hat{\xi}_2)} - 2\frac{cov(\hat{\xi}_1, \hat{\xi}_2)}{E(\hat{\xi}_1)E(\hat{\xi}_2)}\right\}. \quad (8)$$

Because information on covariance between the smaller and larger samples was unavailable from the selected studies, we take into account that the larger sample P_1 is a superposition of two independent subsets P_2 and P_0, where $P_0 = P_1 - P_2$. Then, the effect size for P_1 can be defined as mean of the effect sizes in these two subsets, i.e. $\hat{b}_1 = \left[\hat{b}_2 \cdot N_2 + \Delta\hat{b} \cdot (N_1 - N_2)\right]/N_1$. Using equation (4) one can derive the following expression for covariance

$$cov(\hat{\xi}_1, \hat{\xi}_2) = var(\hat{\xi}_2)\frac{N_2}{N_1}\frac{\sqrt{E(\hat{\xi}_1) + \frac{\ln\alpha}{N_1}}}{\sqrt{E(\hat{\xi}_2) + \frac{\ln\alpha}{N_2}}}, \quad (9)$$

The 95% confidence intervals (CIs) for ρ evaluated using (8,9) are given in the last two columns in Supplementary Tables 1-4. Hypothesis H1 should be rejected if value for $\hat{\rho}$ is not within the 95% CIs.

We also used bootstrapping to generate 95% CIs for $\hat{\rho} = \hat{\xi}_1/\hat{\xi}_2$ to reject hypothesis H1. For each locus in Supplementary Tables 1-4, we generated a mega sample ($N=10^7$) of two random variables X and Y. Variable X was distributed as a standard normal variate with variance 1 and zero mean. Variable Y was constructed as dependent variable in the regression model $Y \sim \alpha + \beta X + \varepsilon$ with intercept α, effect size β, and residual $\varepsilon \sim N(0, sd_\varepsilon)$. To simulate statistics for $\hat{\rho} = \hat{\xi}_1/\hat{\xi}_2$, standard deviation for ε was defined based on a larger sample P_1 of size N_1 ($\hat{\xi}_1$), $sd_\varepsilon = sd_{\hat{\xi}_1} = \sqrt{N_1}\beta/\Phi^{-1}(p_1)$, where Φ^{-1} is the quantile function for normal distribution and

p_1 denotes probability for sample P_1. Next we randomly selected sample P_1 from the mega sample. Given that larger and smaller samples in the GWAS selected for our study are not independent, we randomly generated a smaller sample P_2 of size N_2 from the larger sample P_1. For simplicity we fixed $\alpha = 1$ and $\beta = 0.1$ in the regression model for all simulations; this does not violate generalizability of the method. Next we conducted linear regression analysis to generate p-values, ξ_1, and ξ_2 for the selected samples P_1 and P_2. Given ξ_1, and ξ_2, we evaluated $\rho = \xi_1/\xi_2$. We conducted 100 and 1000 simulations for each locus to generate c.d.f. for $\hat{\rho}$ and to evaluate 95% CIs. We did not conduct more simulations because c.d.f. asymptotically converged. The 95% CIs for ρ evaluated using this method for 1000 simulations confirm our analytical result given in the last two columns in Supplementary Tables 1-4.

2. Supplementary Tables

Supplementary Table 1

P-value difference and the relative efficiency of the BMI GWAS

N	Locus	CHR	Nature Genetics 2010			Nature 2015			Δp	ρ	*95% CI dep*	
			SNP	N	*p*-value	SNP	N	*p*-value			lower	upper
1	FTO	16	rs1558902	192,344	4.75E-120	rs1558902	320,073	7.5E-153	32.80	0.77	0.917	1.083
2	SEC16B	1	rs543874	179,414	3.56E-23	rs543874	322,008	2.62E-35	12.13	0.86	0.773	1.227
3	MC4R	18	rs571312	203,600	6.43E-42	rs6567160	321,958	3.93E-53	11.20	0.81	0.857	1.143
4	TFAP2B	6	rs987237	195,776	2.90E-20	rs2207139	322,019	4.13E-29	8.85	0.88	0.766	1.234
5	GNPDA2	4	rs10938397	197,008	3.78E-31	rs10938397	320,955	3.21E-38	7.07	0.76	0.838	1.162
6	FAIM2	12	rs7138803	200,064	1.82E-17	rs7138803	322,092	8.15E-24	6.35	0.86	0.755	1.245
7	MTCH2	11	rs3817334	191,943	1.59E-12	rs3817334	321,959	5.15E-17	4.49	0.82	0.709	1.291
8	SCG3	15	rs2652594	238,618	7.67E-05	rs3736485	321,398	7.41E-09	4.01	1.47	0.197	1.803
9	ETV5	3	rs9816226	196,221	1.69E-18	rs1516725	320,644	1.89E-22	3.95	0.75	0.788	1.212
10	NEGR1	1	rs2815752	198,380	1.61E-22	rs3101336	316,872	2.66E-26	3.78	0.73	0.817	1.183
11	FLJ35779	5	rs2112347	231,729	2.17E-13	rs2112347	322,019	6.19E-17	3.54	0.92	0.740	1.260
12	HNF4G	8	rs2922763	207,807	6.46E-08	rs17405819	322,085	2.07E-11	3.49	0.96	0.584	1.416
13	CADM2	3	rs13078807	237,404	3.94E-11	rs13078960	322,135	1.74E-14	3.36	0.98	0.702	1.298
14	NRXN3	14	rs10150332	183,022	2.75E-11	rs7141420	321,970	1.23E-14	3.35	0.75	0.706	1.294
15	C9orf4	9	rs6477694	234,158	4.84E-05	rs6477694	322,048	2.67E-08	3.26	1.28	0.324	1.676
16	SH2B1	16	rs7359397	204,309	1.88E-20	rs3888190	321,930	3.14E-23	2.78	0.72	0.811	1.189
17	TMEM160	19	rs3810291	233,512	1.64E-12	rs3810291	296,261	4.81E-15	2.53	0.96	0.755	1.245
18	PTBP2	1	rs1555543	243,013	3.68E-10	rs11165643	320,730	2.07E-12	2.25	0.94	0.710	1.290
19	NUDT3	6	rs206936	249,777	3.02E-08	rs205262	315,542	1.75E-10	2.24	1.03	0.667	1.333
20	RPL27A	11	rs4929949	249,791	2.80E-09	rs4256980	320,028	2.90E-11	1.98	0.96	0.703	1.297
21	BDNF	11	rs10767664	204,158	4.69E-26	rs11030104	322,103	5.56E-28	1.93	0.68	0.842	1.158
22	RBJ	2	rs713586	230,748	6.17E-22	rs10182181	321,759	8.78E-24	1.85	0.78	0.828	1.172
23	KIAA1505	7	rs6955651	125,672	1.92E-06	rs2245368	205,675	3.19E-08	1.78	0.80	0.582	1.418
24	ADCY9	16	rs2444217	198,786	9.49E-08	rs879620	320,000	2.17E-09	1.64	0.77	0.645	1.355
25	QPCTL	19	rs2287019	194,564	1.88E-16	rs2287019	300,921	4.59E-18	1.61	0.71	0.793	1.207
26	TMEM18	2	rs2867125	197,806	2.77E-49	rs13021737	318,287	1.11E-50	1.40	0.64	0.890	1.110
27	TNNI3K	1	rs1514175	227,900	8.16E-14	rs12566985	319,282	3.28E-15	1.40	0.79	0.774	1.226
28	LMX1B	9	rs867559	226,483	1.39E-07	rs10733682	320,727	1.83E-08	0.88	0.80	0.670	1.330

29	MTIF3	13	rs4771122	198,577	9.48E-10	rs12016871	233,803	2.29E-10	0.62	0.91	0.772	1.228
30	LRRN6C	9	rs10968576	216,916	2.65E-13	rs10968576	322,061	6.61E-14	0.60	0.71	0.774	1.226
31	KCTD15	19	rs29941	192,872	3.01E-09	rs29941	321,970	2.41E-08	-0.90	0.54	0.749	1.251
32	SLC39A8	4	rs13107325	245,378	1.50E-13	rs13107325	321,461	1.83E-12	-1.09	0.70	0.807	1.193
33	FANCL	2	rs887912	242,807	1.79E-12	rs1016287	321,969	2.25E-11	-1.10	0.68	0.799	1.201
34	MAP2K5	15	rs2241423	227,950	1.19E-18	rs16951275	322,098	1.91E-17	-1.21	0.66	0.831	1.169
35	PRKD1	14	rs11847697	241,667	5.76E-11	rs11847697	306,243	3.99E-09	-1.84	0.65	0.799	1.201
36	LRP1B	2	rs2890652	209,068	1.35E-10	rs2121279	322,065	2.31E-08	-2.23	0.50	0.788	1.212
37	GPRC5B	16	rs12444979	239,715	2.91E-21	rs12446632	316,758	1.48E-18	-2.71	0.66	0.854	1.146
38	RASA2[*]	3	rs2035935	245,575	5.71E-04	rs16851483	233,929	3.55E-10	-6.21	0.33	0.831	1.169

This table uses the information from Supplementary Tables 1 in Ref.[20] and 4 in Ref.[21]except the results for the ADCY9 locus from Table 3 in Ref.[21].

[*] The sample size for locus RASA2 was larger in the 2010 Nature Genetics study [20] than in the 2015 Nature study [21]. Accordingly, the results for this locus for rs2035935 were taken from Ref.[20] and those for rs16851483 from Ref.[21].

$\Delta p = -(\log_{10}(p_{2015}) - \log_{10}(p_{2010}))$ is the difference in the log-transformed p-values reported in the larger sample [21] and the smaller sample [20].

Parameter $\rho = \xi_1/\xi_2$ is the relative efficiency of the meta-analyses indicating the gain in log-transformed *p*-value per person in the larger sample compared to the smaller sample.

Eight loci below double lines numbered as (#31-38) have larger (smaller) *p*-value in the larger (smaller) sample (negative values in column Δp).

Hypothesis H1 should be rejected if value for $\hat{\rho}$ are not within the 95% CIs. Cells for loci (SNPs) for which hypothesis H1 is rejected are highlighted with grey color.

Supplementary Table 2

P-value difference and the relative efficiency of the BMI GWAS in the selected samples from the Nature Genetics study

N	Locus	CHR	SNP	NG2010		NG2010, Stage 1		Δp	ρ	*95% CI dep*	
				N	*p*-value	N	*p*-value			lower	upper
1	FTO	16	rs1558902	192,344	4.75E-120	123,846	2.05E-62	57.63	1.24	0.824	1.176
2	SEC16B	1	rs543874	179,414	3.56E-23	123,863	1.66E-13	9.67	1.21	0.638	1.362
3	MC4R	18	rs571312	203,600	6.43E-42	123,812	2.14E-22	19.52	1.15	0.709	1.291
4	TFAP2B	6	rs987237	195,776	2.90E-20	123,860	5.97E-16	4.32	0.81	0.760	1.240
5	GNPDA2	4	rs10938397	197,008	3.78E-31	123,849	4.35E-17	14.06	1.17	0.665	1.335
6	FAIM2	12	rs7138803	200,064	1.82E-17	123,799	3.96E-11	6.34	1.00	0.633	1.367
7	MTCH2	11	rs3817334	191,943	1.59E-12	123,815	4.79E-11	1.48	0.74	0.730	1.270
8	**SCG3**	15	rs2652594	238,618	7.67E-05	123,848	3.81E-06	**-1.30**	0.39	0.714	1.286
9	ETV5	3	rs9816226	196,221	1.69E-18	123,859	7.61E-14	4.65	0.85	0.728	1.272
10	NEGR1	1	rs2815752	198,380	1.61E-22	123,849	1.17E-14	7.86	0.98	0.695	1.305
11	FLJ35779	5	rs2112347	231,729	2.17E-13	123,863	4.76E-08	5.34	0.92	0.545	1.455
12	HNF4G	8	rs2922763	207,807	6.46E-08	123,861	2.13E-07	0.52	0.64	0.675	1.325
13	CADM2	3	rs13078807	237,404	3.94E-11	123,808	1.06E-07	3.43	0.78	0.595	1.405
14	NRXN3	14	rs10150332	183,022	2.75E-11	123,865	2.03E-07	3.87	1.07	0.536	1.464
15	**C9orf4**	9	rs6477694	234,158	4.84E-05	123,801	5.36E-06	**-0.95**	0.43	0.695	1.305
16	SH2B1	16	rs7359397	204,309	1.88E-20	123,864	2.41E-10	10.11	1.24	0.511	1.489
17	TMEM160	19	rs3810291	233,512	1.64E-12	119,531	1.04E-07	4.81	0.86	0.552	1.448
18	PTBP2	1	rs1555543	243,013	3.68E-10	123,856	7.81E-07	3.32	0.79	0.553	1.447
19	NUDT3	6	rs206936	249,777	3.02E-08	123,865	4.56E-06	2.18	0.70	0.561	1.439
20	RPL27A	11	rs4929949	249,791	2.80E-09	123,860	7.57E-08	1.43	0.59	0.679	1.321
21	BDNF	11	rs10767664	204,158	4.69E-26	123,865	5.53E-13	13.07	1.25	0.568	1.432
22	RBJ	2	rs713586	230,748	6.17E-22	123,718	2.51E-07	14.61	1.72	0.060	1.940
23	**KIAA1505**	7	rs6955651	125,672	1.92E-06	82,760	1.68E-06	**-0.05**	0.65	0.665	1.335
24	ADCY9	16	rs2444217	198,786	9.49E-08	123,855	3.44E-06	1.56	0.80	0.575	1.425
25	QPCTL	19	rs2287019	194,564	1.88E-16	100,473	3.18E-07	9.23	1.25	0.318	1.682
26	TMEM18	2	rs2867125	197,806	2.77E-49	123,833	4.93E-22	27.25	1.42	0.639	1.361
27	TNNI3K	1	rs1514175	227,900	8.16E-14	123,835	1.41E-09	4.24	0.80	0.646	1.354
28	LMX1B	9	rs867559	226,483	1.39E-07	123,779	2.24E-06	1.21	0.66	0.615	1.385
29	MTIF3	13	rs4771122	198,577	9.48E-10	123,861	1.20E-07	2.10	0.81	0.626	1.374

30	LRRN6C	9	rs10968576	216,916	2.65E-13	123,861	1.88E-08	4.85	0.93	0.572	1.428
31	**KCTD15**	19	rs29941	192,872	3.01E-09	123,842	2.42E-09	**-0.10**	0.64	0.731	1.269
32	SLC39A8	4	rs13107325	245,378	1.50E-13	123,348	1.37E-07	5.96	0.94	0.502	1.498
33	FANCL	2	rs887912	242,807	1.79E-12	123,855	2.69E-06	6.18	1.08	0.356	1.644
34	MAP2K5	15	rs2241423	227,950	1.19E-18	123,835	1.15E-10	7.98	0.98	0.596	1.404
35	PRKD1	14	rs11847697	241,667	5.76E-11	119,851	1.11E-08	2.29	0.64	0.677	1.323
36	LRP1B	2	rs2890652	209,068	1.35E-10	123,855	2.38E-07	3.25	0.88	0.569	1.431
37	GPRC5B	16	rs12444979	239,715	2.91E-21	123,865	4.20E-11	10.16	1.02	0.577	1.423
38	**RASA2**	3	rs2035935	245,575	5.71E-04	123,787	1.08E-06	**-2.73**	0.27	0.765	1.235

This table uses the information from Supplementary Table 1 in Ref.[20].
NG2010 denotes the entire sample in Ref.[20].
NG2010, Stage 1 denotes the sample from Stage 1 in Ref.[20].
$\Delta p = -(\log_{10}(p_{2010}) - \log_{10}(p_{2010\_St1}))$ is the difference in the log-transformed p-values reported in the larger sample (NG2010) and the smaller sample (NG2010, Stage 1).
Parameter $\rho = \xi_1/\xi_2$ is the relative efficiency indicating the gain in log-transformed p-value per person in the larger sample compared to the smaller sample.
Five loci (bolded font) have larger (smaller) *p*-value in the larger (smaller) sample (see column Δp).
The order of loci is the same as in Supplementary Table 1.
Hypothesis H1 should be rejected if value for $\hat{\rho}$ are not within the 95% CIs. Cells for loci (SNPs) for which hypothesis H1 is rejected are highlighted with grey color.

Supplementary Table 3

P-value difference and the relative efficiency of the BMI GWAS in the selected samples from the Nature study

N	Locus	CHR	SNP	N2015		N2015, Metabochip		Δ*p*	*ρ*	*95% CI dep*	
				N	*p*-value	N	*p*-value			lower	upper
1	FTO	16	rs1558902	320,073	7.5E-153	86,411	3.60E-49	103.68	0.85	0.807	1.193
2	SEC16B	1	rs543874	322,008	2.62E-35	88,120	2.19E-10	24.92	0.98	0.481	1.519
3	MC4R	18	rs6567160	321,958	3.93E-53	88,119	2.63E-13	39.82	1.14	0.477	1.523
4	TFAP2B	6	rs2207139	322,019	4.13E-29	88,125	8.03E-12	17.29	0.70	0.655	1.345
5	GNPDA2	4	rs10938397	320,955	3.21E-38	87,941	3.20E-13	25.00	0.82	0.623	1.377
6	FAIM2	12	rs7138803	322,092	8.15E-24	88,115	4.82E-08	15.77	0.86	0.468	1.532
7	MTCH2	11	rs3817334	321,959	5.15E-17	88,063	4.35E-08	8.93	0.61	0.620	1.380
8	SCG3	15	rs3736485	321,398	7.41E-09	88,117	1.55E-04	4.32	0.59	0.452	1.548
9	ETV5	3	rs1516725	320,644	1.89E-22	88,019	8.96E-07	15.68	0.99	0.315	1.685
10	NEGR1	1	rs3101336	316,872	2.66E-26	82,925	8.40E-09	17.50	0.83	0.510	1.490
11	FLJ35779	5	rs2112347	322,019	6.19E-17	88,110	1.09E-04	12.25	1.12	0.554*	1.753*
12	HNF4G	8	rs17405819	322,085	2.07E-11	88,099	6.24E-03	8.48	1.33	0.485*	2.038*
13	CADM2	3	rs13078960	322,135	1.74E-14	88,122	3.83E-08	6.34	0.51	0.679	1.321
14	NRXN3	14	rs7141420	321,970	1.23E-14	88,111	6.17E-05	9.70	0.90	0.222	1.778
15	C9orf4	9	rs6477694	322,048	2.67E-08	88,091	8.93E-04	4.52	0.68	0.269	1.731
16	SH2B1	16	rs3888190	321,930	3.14E-23	87,924	6.94E-11	12.34	0.61	0.682	1.318
17	TMEM160	19	rs3810291	296,261	4.81E-15	63,550	2.62E-05	9.74	0.67	0.428	1.572
18	PTBP2	1	rs11165643	320,730	2.07E-12	88,086	8.52E-04	8.61	1.05	0.463*	1.952*
19	NUDT3	6	rs205262	315,542	1.75E-10	82,938	3.62E-05	5.31	0.58	0.508	1.492
20	RPL27A	11	rs4256980	320,028	2.90E-11	86,027	1.12E-04	6.59	0.72	0.349	1.651
21	BDNF	11	rs11030104	322,103	5.56E-28	88,123	2.12E-09	18.58	0.86	0.516	1.484
22	RBJ	2	rs10182181	321,759	8.78E-24	88,075	3.48E-08	15.60	0.85	0.480	1.520
23	KIAA1505	7	rs2245368	205,675	3.19E-08	36,288	4.82E-02	6.18	1.00	0.323*	3.847*
24	**ADCY9**	16	rs879620	320,000	2.17E-09						
25	QPCTL	19	rs2287019	300,921	4.59E-18	88,097	3.48E-10	7.88	0.54	0.708	1.292
26	TMEM18	2	rs13021737	318,287	1.11E-50	88,035	2.34E-17	33.32	0.83	0.673	1.327
27	TNNI3K	1	rs12566985	319,282	3.28E-15	85,311	1.20E-06	8.56	0.65	0.540	1.460
28	LMX1B	9	rs10733682	320,727	1.83E-08	88,097	1.28E-01	6.84	2.38	0.414*	2.425*
29	**MTIF3**	13	rs12016871	233,803	2.29E-10						

30	LRRN6C	9	rs10968576	322,061	6.61E-14	88,106	4.33E-06	7.82	0.67	0.500	1.500
31	KCTD15	19	rs29941	321,970	2.41E-08	87,975	2.42E-02	6.00	1.29	0.418*	2.479*
32	SLC39A8	4	rs13107325	321,461	1.83E-12	87,985	1.45E-05	6.90	0.66	0.476	1.524
33	FANCL	2	rs1016287	321,969	2.25E-11	88,069	8.86E-04	7.59	0.95	0.000	2.022
34	MAP2K5	15	rs16951275	322,098	1.91E-17	88,118	6.25E-06	11.51	0.88	0.334	1.666
35	PRKD1	14	rs11847697	306,243	3.99E-09	87,878	3.05E-03	5.88	0.96	0.442*	2.352*
36	LRP1B	2	rs2121279	322,065	2.31E-08	88,099	2.04E-03	4.95	0.78	0.082	1.918
37	GPRC5B	16	rs12446632	316,758	1.48E-18	82,754	1.03E-05	12.84	0.93	0.271	1.729
38	**RASA2**	3	rs16851483	233,929	3.55E-10						

This table uses the information from Supplementary Table 4 in Ref.[21] except the results for the ADCY9 locus from Table 3 in Ref.[21].
N2015 denotes the entire sample in Ref.[21] .
N2015, Metabochip denotes the sample from Metabochip in Ref.[21].
$\Delta p = -(\log_{10}(p_{2015}) - \log_{10}(p_{2015\_MCH}))$ is the difference in the log-transformed p-values reported in the larger sample (N2015) and the smaller sample (N2015, Metabochip).
Parameter $\rho = \xi_1/\xi_2$ is the relative efficiency indicating the gain in log-transformed p-value per person in the larger sample compared to the smaller sample.
The results using Metabochip were lacking for three loci shown in bold.
The order of loci is the same as in Supplementary Table 1.
Hypothesis H1 should be rejected if value for $\hat{\rho}$ are not within the 95% CIs. Cells for loci (SNPs) for which hypothesis H1 is rejected are highlighted with grey color.
[*] For these loci t_i values were not in the range of the approximations for the developed approach (Supplementary Note 1, Figure). Accordingly, for these loci we used the results of numerical simulations as described in Supplementary Note 1.

Supplementary Table 4

P-value difference and the relative efficiency of the lipid GWAS

N	Trait	Locus	CHR	Nature 2010				Nature Genetics 2013				Δ*p*	*ρ*	*95% CI dep*	
				SNP	N	*p*-value	Ref	SNP	N	*p*-value	Ref			lower	upper
1	HDL	CETP	16	rs3764261	94,225	7.10e-380	ST2	rs3764261	177,533	1.4e-769	ST12B	211.11	1.08		
2	LDL	LDLR	19	rs6511720	93,131	4.28E-117	ST2	rs6511720	170,607	3.9E-262	ST12B	145.04	1.23	0.858	1.142
3	TG	GCKR	2	rs1260326	96,590	5.68E-133	ST2	rs1260326	177,765	2.3E-239	ST12E	106.42	0.98	0.894	1.106
4	HDL	LIPC	15	rs1532085	98,409	2.92E-96	ST2	rs1532085	185,482	1.2E-188	ST12E	92.39	1.04	0.866	1.134
5	TG	LPL	8	rs12678919	96,598	1.50E-115	ST2	rs12678919	177,749	1.8E-199	ST12B	83.92	0.94	0.891	1.109
6	LDL	SORT1	1	rs629301	95,454	9.70E-171	ST2	rs629301	142,643	5.4E-241	ST12B	70.25	0.95	0.923	1.077
7	LDL	APOB	2	rs1367117	95,446	4.48E-114	ST2	rs1367117	173,007	9.5E-183	ST12B	68.67	0.89	0.897	1.103
8	TG	TRIB1	8	rs2954029	96,598	3.29E-55	ST2	rs2954029	177,729	1.0E-107	ST12B	52.52	1.07	0.819	1.181
9	TG	MLXIPL	7	rs17145738	97,000	6.00E-58	T1	rs17145738	176,000	9.0E-99	ST3	40.82	0.94	0.846	1.154
10	TC	ANGPTL3	1	rs3850634	97,148	4.90E-41	ST2	rs2131925	187,000	4.0E-80	ST3	39.09	1.02	0.793	1.207
11	TC	CILP2	19	rs10401969	98,640	2.90E-38	ST2	rs10401969	185,666	4.1E-77	ST12B	38.85	1.08	0.776	1.224
12	LDL	HMGCR	5	rs12916	95,454	5.12E-45	ST2	rs12916	168,000	8.0E-78	ST3	32.81	0.99	0.819	1.181
13	HDL	ABCA1	9	rs1883025	99,179	1.75E-33	ST2	rs1883025	186,365	1.5E-65	ST12B	32.07	1.05	0.766	1.234
14	LDL	APOE	19	rs4420638	83,209	8.72E-147	ST2	rs4420638	93,103	1.5E-178	ST12B	31.76	1.09	0.946	1.054
15	LDL	ABO	9	rs9411489	96,000	6.00E-13	T1	rs9411489	119,312	1.8E-41	ST12F	28.52	2.68	0.164	1.836
16	TC	ABCG5/8	2	rs4299376	95,992	4.03E-45	ST2	rs4299376	158,000	3.0E-73	ST3	28.13	0.99	0.828	1.172
17	LDL	PCSK9	1	rs2479409	95,435	1.93E-28	ST2	rs2479409	172,970	2.5E-50	ST12B	21.89	0.99	0.765	1.235
18	HDL	LCAT	16	rs16942887	98,409	8.39E-33	ST2	rs16942887	186,000	8.0E-54	ST3	21.02	0.88	0.801	1.199
19	HDL	GALNT2	1	rs4846914	99,881	3.66E-21	ST2	rs4846914	186,995	3.5E-41	ST12B	20.02	1.06	0.699	1.301
20	HDL	HNF4A	20	rs1800961	71,749	1.05E-15	ST2	rs1800961	157,871	1.6E-34	ST12F	18.82	1.03	0.628	1.372
21	LDL	HPR	16	rs2000999	93,999	1.75E-22	ST2	rs2000999	172,000	4.0E-41	ST3	18.64	1.01	0.727	1.273
22	LDL	FADS1-2-3	11	rs174583	95,443	1.17E-21	ST2	rs174546	173,000	2.0E-39	ST3	17.77	1.02	0.720	1.280
23	HDL	SCARB1	12	rs838880	80,428	2.58E-14	ST2	rs838880	173,000	6.0E-32	ST3	17.63	1.07	0.596	1.404
24	HDL	PLTP	20	rs6065906	98,409	1.90E-22	ST2	rs6065906	186,000	5.0E-40	ST3	17.58	0.96	0.735	1.265
25	HDL	PPP1R3B	8	rs9987289	99,900	6.40E-25	ST2	rs9987289	169,235	2.0E-41	ST12E	16.51	0.99	0.759	1.241

26	TC	TIMD4	5	rs6882076	100,184	7.46E-28	ST2	rs6882076	187,000	5.0E-41	ST3	13.17	0.80	0.803	1.197
27	HDL	LRP4	11	rs3136441	99,900	3.48E-18	ST2	rs3136441	186,975	6.8E-29	ST12A	10.71	0.86	0.735	1.265
28	HDL	ZNF648	1	rs1689800	99,900	3.18E-10	ST2	rs1689800	187,000	5.0E-20	ST3	9.80	1.09	0.533	1.467
29	HDL	UBE2L3	22	rs181362	96,905	1.11E-08	ST2	rs181362	178,283	4.3E-18	ST12A	9.41	1.19	0.440	1.560
30	HDL	CMIP	16	rs2925979	98,409	2.09E-11	ST2	rs2925979	185,553	1.3E-19	ST12C	8.21	0.94	0.622	1.378
31	HDL	IRS1	2	rs2972146	97,000	3.00E-09	T1	rs2972146	184,044	1.9E-17	ST12A	8.20	1.03	0.529	1.471
32	HDL	PABPC4	1	rs4660293	99,855	3.99E-10	ST2	rs4660293	187,027	2.9E-18	ST12C	8.14	1.00	0.570	1.430
33	HDL	LOC55908	19	rs737337	98,409	3.10E-09	ST2	rs737337	185,432	4.6E-17	ST12C	7.83	1.02	0.535	1.465
34	HDL	TTC39B	9	rs581080	100,000	3.00E-12	T1	rs581080	186,937	1.0E-19	ST12G	7.48	0.88	0.662	1.338
35	HDL	LILRA3	19	rs386000	86,430	4.29E-16	ST2	rs386000	165,000	3.0E-23	ST3	7.16	0.77	0.741	1.259
36	TC	DNAH11	7	rs12670798	100,000	9.00E-10	T1	rs12670798	187,287	9.5E-17	ST12E	6.98	0.95	0.583	1.417
37	TG	KLHL8	4	rs442177	96,598	8.65E-12	ST2	rs442177	177,798	1.3E-18	ST12B	6.82	0.88	0.657	1.343
38	TC	LPA	6	rs1564348	100,168	9.71E-17	ST2	rs1564348	187,000	3.0E-23	ST3	6.51	0.75	0.756	1.244
39	HDL	UBASH3B	11	rs7115089	99,900	2.66E-08	ST2	rs7941030	187,000	1.0E-14	ST3	6.42	0.99	0.520	1.480
40	TC	FLJ36070	19	rs492602	97,148	2.01E-10	ST2	rs492602	184,180	1.1E-16	ST12A	6.26	0.87	0.629	1.371
41	HDL	TRPS1	8	rs2293889	99,900	5.77E-11	ST2	rs2293889	180,102	4.3E-17	ST12A	6.13	0.89	0.643	1.357
42	LDL	MYLIP	6	rs3757354	91,293	1.16E-11	ST2	rs3757354	172,986	2.1E-17	ST12G	5.74	0.80	0.680	1.320
43	TG	MAP3K1	5	rs9686661	95,848	1.32E-10	ST2	rs9686661	177,050	2.5E-16	ST12B	5.72	0.85	0.647	1.353
44	TC	GPAM	10	rs2255141	100,184	2.03E-10	ST2	rs2255141	187,266	6.5E-16	ST12F	5.49	0.84	0.644	1.356
45	LDL	HNF1A	12	rs1169288	95,454	1.13E-15	ST2	rs1169288	163,000	6.0E-21	ST3	5.27	0.79	0.749	1.251
46	LDL	ST3GAL4	11	rs11220462	95,454	1.20E-15	ST2	rs11220462	145,030	6.6E-21	ST12B	5.26	0.89	0.744	1.256
47	TC	RAB3GAP1	2	rs7570971	95,000	2.00E-08	T1	rs7570971	184,956	1.2E-13	ST12A	5.22	0.86	0.577	1.423
48	HDL	COBLL1	2	rs12328675	99,892	2.72E-10	ST2	rs12328675	187,092	2.1E-15	ST12A	5.11	0.82	0.649	1.351
49	HDL	SLC39A8	4	rs13107325	92,059	7.20E-11	ST2	rs13107325	179,316	1.1E-15	ST12A	4.82	0.76	0.677	1.323
50	LDL	NPC1L1	7	rs217386	95,454	4.25E-11	ST2	rs2072183	170,000	7.0E-16	ST3	4.78	0.82	0.675	1.325
51	TC	BRAP	12	rs11065987	100,184	6.77E-12	ST2	rs11065987	187,309	2.1E-16	ST12A	4.51	0.75	0.704	1.296
52	LDL	NYNRIN	14	rs8017377	96,000	5.00E-11	T1	rs8017377	173,000	3.0E-15	ST3	4.22	0.78	0.686	1.314
53	TC	C6orf106	6	rs2814982	100,184	4.68E-11	ST2	rs2814982	187,263	3.7E-15	ST12A	4.10	0.75	0.691	1.309
54	TG	MSL2L1	3	rs645040	96,597	2.52E-08	ST2	rs645040	178,000	2.0E-12	ST3	4.10	0.84	0.596	1.404
55	LDL	HFE	6	rs1800562	93,821	6.07E-10	ST2	rs1800562	171,209	8.3E-14	ST12C	3.86	0.78	0.663	1.337

56	LDL	LDLRAP1	1	rs12027135	95,454	1.24E-10	ST2	rs12027135	165,000	2.0E-14	ST3	3.79	0.80	0.680	1.320
57	TG	LRP1	12	rs11613352	96,598	4.43E-10	ST2	rs11613352	177,799	9.4E-14	ST12A	3.67	0.76	0.672	1.328
58	HDL	STARD3	17	rs11869286	99,000	1.00E-13	T1	rs11869286	177,918	2.7E-17	ST12E	3.57	0.71	0.745	1.255
59	TC	SPTY2D1	11	rs10128711	100,000	3.00E-08	T1	rs10128711	157,199	1.1E-11	ST12B	3.44	0.93	0.600	1.400
60	TG	CYP26A1	10	rs2068888	96,598	2.38E-08	ST2	rs2068888	177,712	1.7E-11	ST12B	3.15	0.77	0.628	1.372
61	TG	CAPN3	15	rs2412710	86,707	1.87E-08	ST2	rs2412710	153,909	1.7E-11	ST12B	3.04	0.79	0.630	1.370
62	LDL	MAFB	20	rs2902941	93,999	1.11E-08	ST2	rs2902940	172,000	2.0E-11	ST3	2.74	0.74	0.651	1.349
63	TG	PINX1	8	rs11776767	96,598	1.30E-08	ST2	rs11776767	177,360	2.9E-11	ST12B	2.65	0.73	0.653	1.347
64	TC	HLA	6	rs3177928	100,151	3.96E-19	ST2	rs3177928	180,000	1.0E-21	ST3	2.60	0.63	0.809	1.191
65	HDL	PGS1	17	rs4129767	99,000	8.00E-09	T1	rs4129767	185,469	2.1E-11	ST12A	2.58	0.70	0.667	1.333
66	HDL	LACTB	15	rs2652834	98,409	8.75E-09	ST2	rs2652834	185,613	3.6E-11	ST12C	2.39	0.69	0.669	1.331
67	HDL	ABCA8	17	rs4148008	98,409	1.79E-10	ST2	rs4148008	165,732	1.1E-12	ST12B	2.21	0.73	0.707	1.293
68	HDL	ARL15	5	rs6450176	99,900	4.98E-08	ST2	rs6450176	187,132	6.9E-10	ST12A	1.86	0.68	0.655	1.345
69	HDL	KLF14	7	rs4731702	99,900	1.21E-15	ST2	rs4731702	187,085	4.8E-17	ST12B	1.40	0.58	0.796	1.204
70	TC	ERGIC3	20	rs2277862	98,656	3.82E-10	ST2	rs2277862	185,738	5.3E-11	ST12G	0.86	0.58	0.737	1.263
71	HDL	PDE3A	12	rs7134375	99,900	3.84E-08	ST2	rs7134375	187,088	1.1E-08	ST12A	0.54	0.57	0.704	1.296
72	TG	PLA2G6	22	rs5756931	95,067	3.82E-08	ST2	rs5756931	174,000	3.0E-08	ST3	0.10	0.55	0.714	1.286
73	HDL	AMPD3	11	rs2923084	99,898	4.62E-08	ST2	rs2923084	187,000	5.0E-08	ST3	-0.03	0.53	0.717	1.283
74	HDL	MC4R	18	rs12967135	98,409	6.58E-09	ST2	rs12967135	153,533	3.6E-08	ST12A	-0.74	0.58	0.742	1.258
75	TC	MOSC1	1	rs2642442	100,000	6.00E-13	T1	rs2642442	111,000	3.0E-11	ST3	-1.70	0.78	0.853	1.147
76	TG	TYW1B	7	rs13238203	78,797	1.13E-09	ST2	rs13238203	101,951	3.1E-06	ST12C	-3.44	0.48	0.793	1.207

This table uses the information from Table 1 (T1) and Supplementary Table 2 (ST2) in Ref.[19] as well as from Supplementary Tables 3 (ST3) and 12A-G (ST12A-G) in Ref.[16].

$\Delta p = -(\log_{10}(p_{2013}) - \log_{10}(p_{2010}))$ is the difference in the log-transformed p-values reported in the larger [16] and smaller [19] samples.

Parameter $\rho = \xi_1/\xi_2$ is the relative efficiency indicating the gain in log-transformed p-value per person in the larger sample compared to the smaller one.

Four loci below double lines (#73-76) have larger (smaller) *p*-value in the larger (smaller) sample (see column Δp).

Hypothesis H1 should be rejected if value for $\hat{\rho}$ are not within the 95% CIs. Cells for loci (SNPs) for which hypothesis H1 is rejected are highlighted with grey color.

3. Supplementary Figures

Supplementary Figure 1

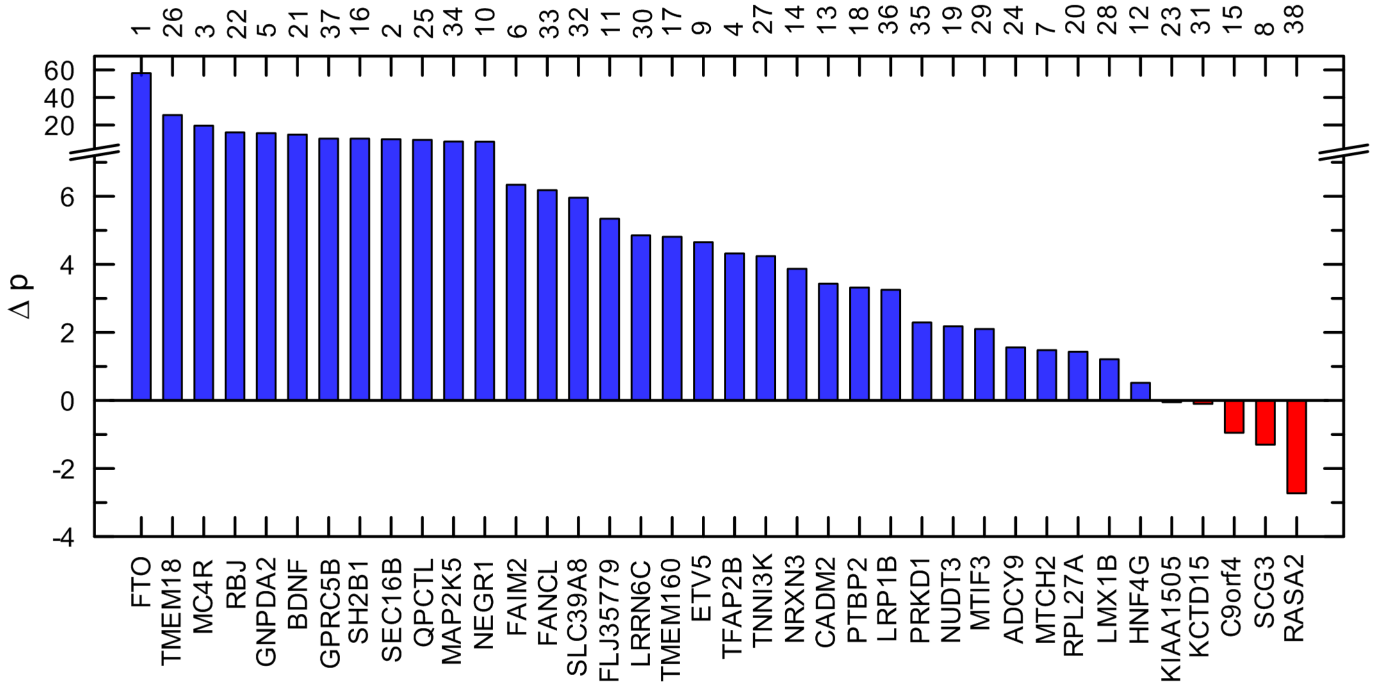

P-value gain in the Nature Genetics GWAS of BMI.

The y-axis shows the difference in log-transformed p-values reported for the entire sample (p_{2010}) and for Stage 1 (p_{2010_St1}) in Ref.[20], i.e., $\Delta p = -(\log_{10}(p_{2010}) - \log_{10}(p_{2010\_St1}))$. The lower x-axis denotes 38 loci selected for the analyses. Numbers on the upper x-axis show the order of loci in Figure 1. Red color ($\Delta p < 0$) shows loci for which p-values were larger in the larger sample compared to the smaller one. Other details and numerical estimates are given in Supplementary Table 2.

Supplementary Figure 2

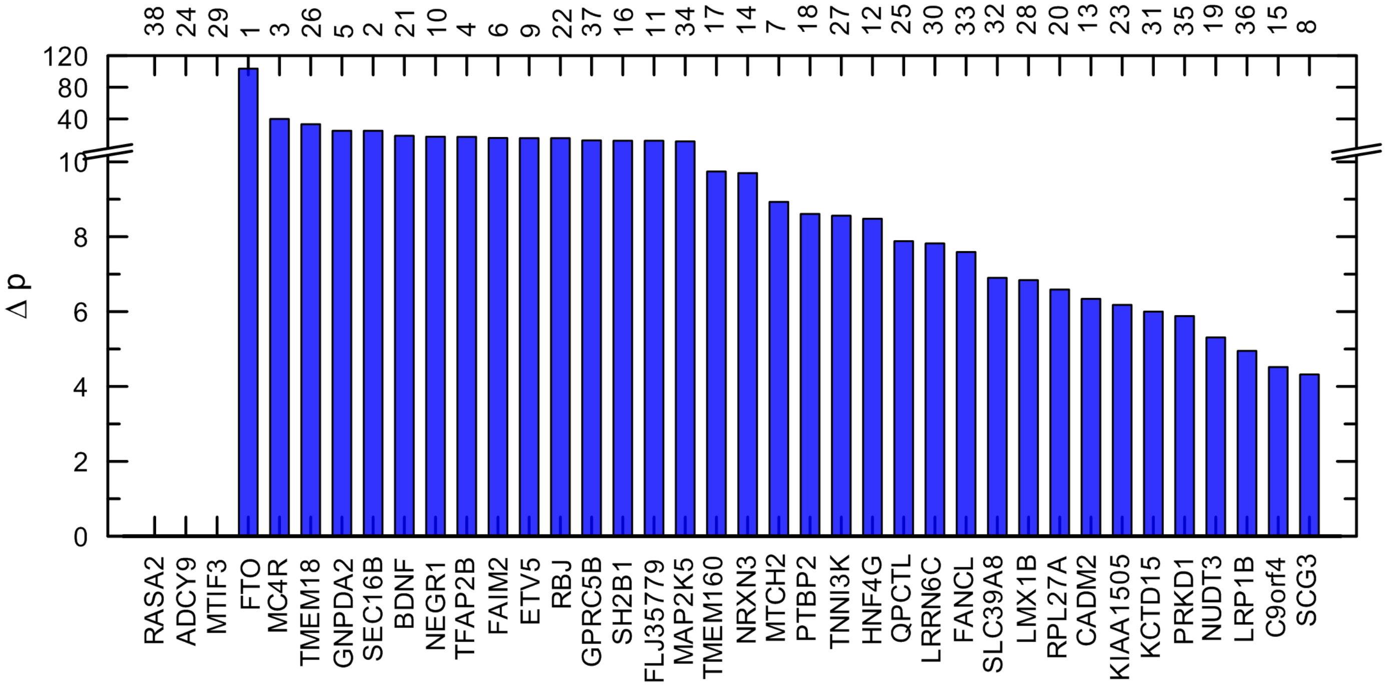

P-value gain in the Nature GWAS of BMI.

The y-axis shows the difference in log-transformed p-values reported for the entire sample (p_{2015}) and for Metabochip (p_{2015_MCH}) in Ref.[21], i.e., $\Delta p = -(\log_{10}(p_{2015}) - \log_{10}(p_{2015\_MCH}))$. The lower x-axis denotes 35 loci available on Metabochip as well as 3 loci which are not available on Metabochip. These loci overlap with those in the Nature Genetics GWAS and, thus, they were selected for the analyses. Numbers on the upper x-axis show the order of loci in Figure 1. Other details and numerical estimates are given in Supplementary Table 3.

Supplementary Figure 3

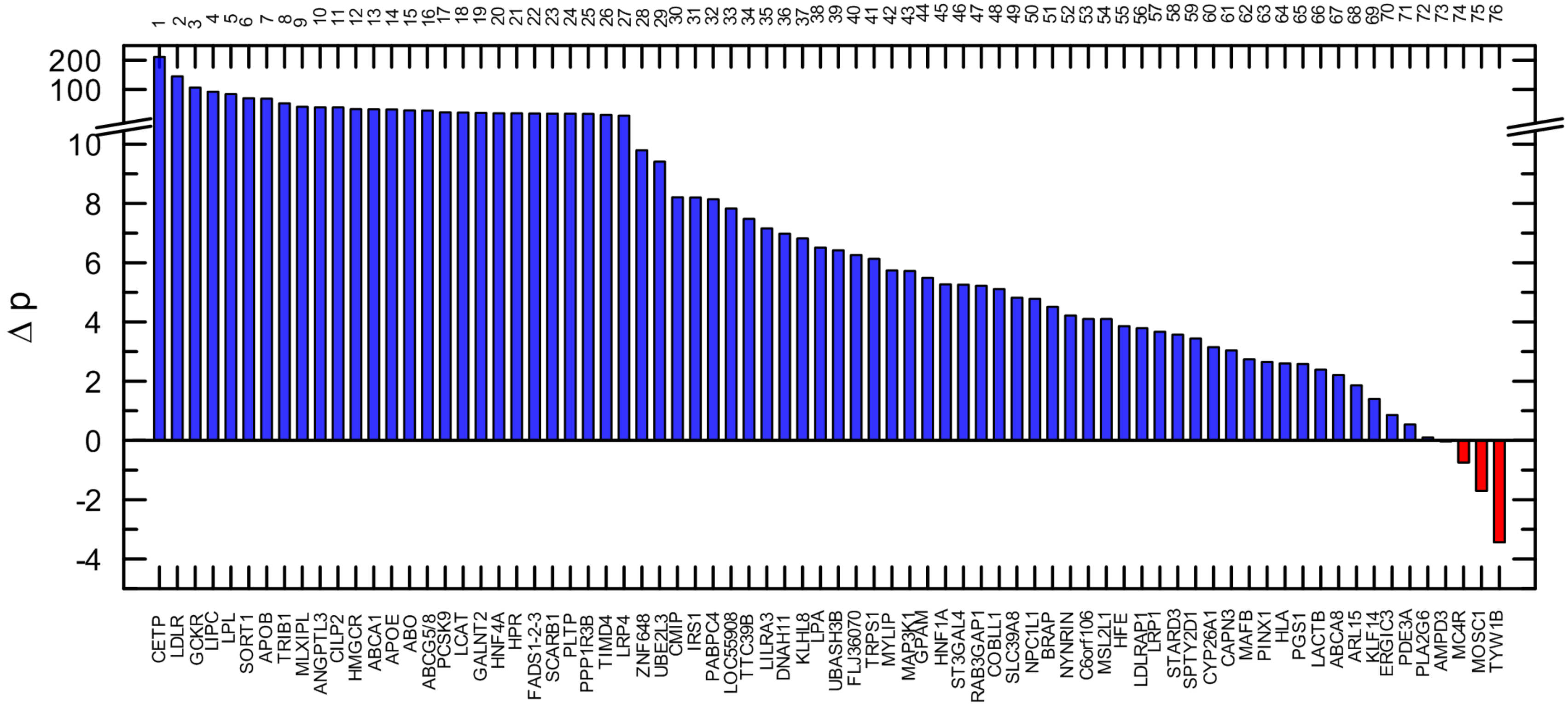

P-value gain in the lipid GWAS.

The y-axis shows the difference in log-transformed p-values reported in larger GWAS (p_{2013}) [16] and smaller GWAS (p_{2010}) [19], i.e., $\Delta p = -(\log_{10}(p_{2013}) - \log_{10}(p_{2010}))$. The lower x-axis denotes 76 loci selected for the analyses. The upper x-axis shows the order of loci. Red color ($\Delta p < 0$) shows loci for which p-values were larger in the larger sample compared to the smaller one. Other details and numerical estimates are given in Supplementary Table 4.
